# Effectiveness of a Positive Youth Development Program for Secondary 1 Students in Macau: A Pilot Study

**DOI:** 10.1100/tsw.2011.97

**Published:** 2011-05-26

**Authors:** Andrew L. Luk, Annah M. L. Au, K. M. Leong, Michelle M. X. Zhu, G. B. Lau, Tammy C. P. Wong, Nancy W. I. Lei

**Affiliations:** Kiang Wu Nursing College of Macau, Macau, China

**Keywords:** adolescents, positive youth development, P.A.T.H.S., objective outcome evaluation, focus groups

## Abstract

With the rapid change to society after the opening of the gaming licensure by the government and the potential attraction to youth caused by the casinos, a well-tested and comprehensive adolescent development program previously established in Hong Kong was adopted and modified to be used in Macau. It is expected to help our adolescents achieve positive growth and be better prepared for future challenges. The aim of this study is to examine the effectiveness of the modified positive youth development program for Secondary 1 Students in Macau. Specifically, two research questions will be asked: (1) How does the positive youth development program affect positive growth for youth in Macau?; and (2) Is youth growth related to different factors such as gender, age, family financial condition, and parents' marital status? A mixed research method with a quantitative approach using a pre- and post-test pre-experimental design, and a qualitative approach using a focus group for the participants is carried out. The study sample included 232 Secondary 1 Students in two schools. The objective outcome evaluation showed that, overall, 123 (53%) of the participants had significant improvement on the total scores of the Chinese Positive Youth Development Scale (CPYDS) and the two composite scores. However, there were some increases in the behavioral intention of alcohol drinking and participation in gambling activities. The “happiness of the family life” was found to have significant differences in the score of the CPYDS, which was shown to be the factor related to youth growth. The focus group interviews revealed that both positive and negative feedback was obtained from the discussion; however, the majority of the participants perceived benefits to themselves from the program. With reference to the principle of triangulation, the present study suggests that, based on both quantitative and qualitative evaluation findings, it should be concluded that there is positive evidence supporting the effectiveness of the Tier 1 Program of the Hong Kong Project P.A.T.H.S. (Positive Adolescent Training through Holistic Social Programmes), which was adopted and modified for Macau. In addition, special attention should be paid to the behavioral intention of alcohol drinking and participation in gambling activities in the local context.

## INTRODUCTION

With the recent economic boom in Macau due to the opening of the gaming licensure by the government in 2002, adolescents are "greatly attracted by high pay from casinos with low education level", which may narrow their views when they are doing their career planning in the secondary schools[1]. Therefore, adolescents must be better equipped for their holistic development especially in the face of the rapidly changing society in Macau. A well-structured local program can surely help our adolescents to maintain positive growth and be better prepared for future challenges. However, reviews of all recent youth studies and programs for adolescent positive growth and development in Macau showed that there is a lack of theoretically sound and comprehensive programs[2,3].

As there are many "working" models available in the literature and research on positive youth development for adolescents, there are wide variations in the related definitions and many address different aspects in their programs[4,5]. In this study, positive youth development is simply defined as "the growth, cultivation, and nurturance of developmental assets, abilities, and potentials in adolescents"[6]. A review by Catalano et al.[7] on 77 programs of positive youth development in North America found that only 25 programs were successful in terms of positive changes in some objective outcome indicators. However, 15 positive youth development constructs were identified in one or more of the goals of these successful programs. These constructs included (1) promotion of bonding, (2) cultivation of resilience, (3) promotion of social competence, (4) promotion of emotional competence, (5) promotion of cognitive competence, (6) promotion of behavioral competence, (7) promotion of moral competence, (8) cultivation of self-determination, (9) development of self-efficacy, (10) promotion of spirituality, (11) promotion of beliefs in the future, (12) development of clear and positive identity, (13) recognition for positive behavior, (14) providing opportunities for prosocial involvement, and (15) fostering prosocial norms.

Some research findings showed that adolescents in Hong Kong faced high levels of stress in different psychosocial domains and there is an intensification of adolescent problems that demand a positive youth development program for holistic development in adolescents[8,9,10]. With the financial support from The Hong Kong Jockey Club Charities Trust, through a joint research project consisting of five universities in Hong Kong, a well-tested and comprehensive positive youth development program, "P.A.T.H.S", has been developed[11,12,13,14]. The word "P.A.T.H.S." denotes **P**ositive **A**dolescent **T**raining through **H**olistic **S**ocial Programmes. There are two tiers of programs (Tier 1 and Tier 2) in this project. The Tier 1 Program is a universal positive youth development program in which students in Secondary 1 to 3 participate, normally with 20 h of training in the full program or at least with 10 h of training in the core program in the school year at each grade. In the Tier 1 Program, the 15 positive youth development constructs identified from the existing successful programs[6] were incorporated. These constructs are named as Bonding (BO), Social Competence (SC), Emotional Competence (EC), Cognitive Competence (CC), Behavioral Competence (BC), Moral Competence (MC), Self-Efficacy (SE), Prosocial Norms (PN), Resilience (RE), Self-Determination (SD), Spirituality (SP), Clear and Positive Identity (ID or CPI), Beliefs in the Future (BF), Prosocial Involvement (PI), and Recognition for Positive Behavior (PB). All these constructs emphasized helping students to learn and develop their personal autonomy on moral principles, or to make independent and critical judgments via a happy, healthy, and stimulating teaching and learning process during their period of schooling. The Tier 2 Program is generally provided for at least one-fifth of the students who have greater psychosocial needs at each grade; in other words, it is a selective program[15].

Since "P.A.T.H.S" was developed in Hong Kong and both Hong Kong and Macau share the similar Chinese culture, this positive growth development program was adopted to be used in Macau with the permission obtained from the research team in Hong Kong. The Tier 1 Program of the "P.A.T.H.S" for Secondary 1 Students was modified by the Macau research team so that the content would reflect the local terminology, such as Macau citizens instead of Hong Kong citizens, government structure of Macau Special Administration Region instead of Hong Kong Special Administration Region, and some indigenous heritage and customs to suit the local context. The revised portion was sent to the Hong Kong team for review to ensure the integrity of the originality of the curriculum. With the support from the Education and Youth Affairs Bureau, the Macau version of the Tier 1 Program of the "P.A.T.H.S" for Secondary 1 Students was piloted in two secondary schools in Macau. The 10-h core program of the Tier 1 Program was completed. Evaluation was conducted after the completion of the piloted project to investigate its effectiveness. Beyond the evaluation of the program, other factors affecting youth growth will also be examined.

The aim of this study is to examine the effectiveness of the positive youth development program for Secondary 1 Students in Macau. Two research questions will be specifically asked:

1. How does the positive youth development program affect the positive growth for youth in Macau?
2. Is youth growth related to different factors such as gender, age, family financial condition, and parents' marital status?

Since there is no theoretically sound and comprehensive program in Macau, a well-structured, comprehensive, positive youth development program is urgently needed to prepare our youth to face the rapidly changing society. If the studied program is effective, it can be used as a complimentary course to our formal courses in school to help our youth to maintain holistic growth.

## METHODS

A mixed research method for the triangulation of data was adopted. A quantitative approach using a pre- and post-test pre-experimental design, and a qualitative approach using a focus group for the participants were adopted.

### Participants and Procedures

The study sample included all Secondary 1 Students in the two schools who chose to join the project after a briefing session conducted by the research team. School A is of a Christian background with a larger student population size. Most of the parents are from the middle social class, with their children of average to better academic performance when compared with School B. School B is of a nonreligious background with a smaller student population. Most of the parents are from the middle to lower social classes, with children of average to weaker academic performance when compared with School A. The average age of students in School B is greater than that of School A since some of them have stayed in Primary 6 for an additional year before being promoted to Secondary 1 or some have stayed in Secondary 1 for an additional year before their performances are up to the standard to be promoted to Secondary 2. In this study, the sample size calculation was based on the total scale of the Chinese Positive Youth Development Scale (CPYDS) developed by Shek et al.[16]. A pre- and post-test one group design was used, and the expected effect size was around 0.22. Then a sample size of 166 (each school = 83) students for the study was needed[17]. The proposed sample size was adequate for this study to achieve 80% power and 5% significance level. The program was conducted by program implementers, including teachers and school social workers, who had been trained for 3 days either in Hong Kong or Macau in order to familiarize themselves with the curriculum and ways of conducting the program.

The two main sources of data included self-reported questionnaires and focus group discussions. The two approaches complemented each other to provide, respectively, an objective and a subjective assessment of the effectiveness of the program. Data were collected at two time points. The first one was before the program started. The pretest self-reported questionnaire was conducted within 1–2 weeks after the start of the school year. The second data collection time point occurred after the students finished the program. Before the start of the project, the purpose of the study was fully explained to the participants by the program implementers and confidentiality of the data collected was repeatedly emphasized. Response to the questionnaire was in a self-administration format and on a voluntary basis. Adequate time was provided for the participants to complete the questionnaire.

Regarding the focus group discussion, one group interview was conducted for both pilot schools after the completion of the program. Ten program participants from School A and six from School B were randomly selected by the program implementers to join the focus group. The aim of randomly selecting participants helped to enhance the generalizability of the findings. The interviews were arranged in the respective schools without the attendance of any teachers or program implementers in order to allow comfort and free expression of feelings and opinions about the program. The time for the interviews took about 1-h each. Since the time was limited, the interview focused the discussion on the general impression and the perceived benefits of the program. The principal investigator conducted the focus group interview with references from the semi-structured interview guide provided by the Hong Kong research team.

Questions regarding general impression of the program include

1. What is your general impression about the program? How do you feel about it?
2. All in all, do you enjoy participating in the program?
3. Are there any activities that can arouse your interest to be involved in the program?
4. Relating to the program, what do you like and what do you dislike?

Questions concerning the perceived benefits of the program include

1. Do you feel that the program is beneficial to the development of adolescents?
2. Do you think that the program has helped your development?
3. After participating in the program, do you have any changes? If yes, please specify.

The content of the interviews was audio taped with the consent of the participants. The content was then transcribed by the research assistant and checked for accuracy by the principal investigator. The raw data of the two groups were analyzed together by developing a coding structure. After comparison of all the coding, relevant themes were developed.

## Instruments

At pre- and post-tests, the participants were invited to respond to a questionnaire including measures of positive youth development, life satisfaction, adolescent problem behavior, and demographic information. The same set of questionnaires was used in the pre- and post-tests; the paired-samples t-test was performed to examine the difference between the scales in pre- and post-test. The components in this questionnaire were described below.

### *Chinese Positive Youth Development Scale (CPYDS)*

The CPYDS is a self-administrated questionnaire developed by Shek et al.[16]. It consists of 15 subscales (90 items) that address the 15 constructs of the program. These subscales are BO (6 items), SC (7 items), EC (6 items), CC (6 items), BC (6 items), MC (6 items), SE (7 items), PN (5 items), RE (6 items), SD (5 items), SP (7 items), ID or CPI (7 items), BF (7 items), PI (5 items), and PB (4 items). The instrument had a good reliability ($\alpha = 0.91$) and ranged from 0.55 to 0.85[16].

Beyond the total score based on all 15 subscales measuring the positive development, two composite indices were also formed. A summation of 10 subscales (resilience, social competence, emotional competence, cognitive competence, behavioral competence, moral competence, self-determination, self-efficacy, beliefs in the future, and clear and positive identity) was used to form a personal development score in assessing psychosocial competence and strengths. A combination of five subscales (social, emotional, cognitive, behavioral, and moral competencies) was used to form the domain of psychosocial competence. If the program is effective, the post-test scores will be significantly higher than the pretest scores on the related measures.

### *Life Satisfaction Scale (LIFE)*

Life satisfaction is another important indicator of positive youth development[18]. The 5-item LIFE was developed by Diener et al.[19] to assess one's own global judgment of one's quality of life. The Chinese version was translated by Shek[20] with acceptable psychometric properties. A higher LIFE scale score indicates a higher level of life satisfaction.

### *Behavioral Intention Scale (BI)*

The 5-item scale was used to assess the adolescents' behavioral intention to engage in problem behavior, including drinking, smoking, taking drugs, having sex with others, and participating in gambling[21]. The scale has a good reliability ($\alpha = 0.84$). A higher BI scale score indicates a higher behavioral intention.

## RESULTS

### Demographic Information

A total of 239 and 242 students participated in the pre- and post-test, respectively. Discarding invalid questionnaires because of missing data in several parts, 232 questionnaires were successfully matched with the same student and were usable for analysis. Among them, 165 were from School A and 67 from School B, 145 were males and 87 were females, and the average age of the participants was 13.7. Details can be seen in Table 1.

### The Objective Outcome Evaluation

As shown in Table 2, the findings revealed that there was a small nonsignificant increase in the total score of the CPYDS in School A, but there were significant positive changes in some of the subscales. Positive changes were also found in both Personal Development and Psychosocial Competence composite scores. However, the scores of the other two scales, LIFE and BI, were less so when compared with those of the pretest. In the BI scale, the score of Item 5, about the intention to participate in gambling, increased slightly, but significantly.

Regarding School B, there was a nonsignificant deterioration in the total score of the CPYDS. The only significant positive change was found in the Social Efficacy subscale. On the other hand, the scores of the other two scales, LIFE and BI, worsened when compared with those of the pretest. The score of Item 1 of BI, about the intention of drinking alcohol, significantly increased.

When seeing the participants as a whole, there was a slight nonsignificant deterioration of the total score of CPYDS and the only subscale with significant positive change was Social Competence. Both the scores of the LIFE and BI scales worsened when compared with those of the pretest. There were also significant increases in Items 1 and 5 in the behavioral intention to drink alcohol and to participate in gambling from pre- to post-tests.

**TABLE 1**
**Participant Characteristics of P.A.T.H.S. Secondary 1 Students in Two Schools**

| Variables | School A (n = 165) | School B (n = 67) | Total (n = 232) |
|---|---|---|---|
| | n (%) | n (%) | n (%) |
| Gender | | | |
| Male | 94 (57.0) | 51 (76.1) | 145 (62.5) |
| Female | 71 (43.0) | 16 (23.9) | 87 (37.5) |
| Age | | | |
| 12 | 28 (17.0) | 4 (6.0) | 32 (13.8) |
| 13 | 69 (41.8) | 12 (17.9) | 81 (34.9) |
| 14 | 47 (28.5) | 21 (31.3) | 68 (29.3) |
| 15 | 12 (7.3) | 18 (26.9) | 30 (12.9) |
| >16 | 9 (5.5) | 12 (17.9) | 21 (9.1) |
| Family members | | | |
| 1 | 1 (0.6) | 0 (0.0) | 1 (0.4) |
| 2 | 5 (3.0) | 2 (3.0) | 7 (3.0) |
| 3 | 29 (17.6) | 15 (22.4) | 44 (19.0) |
| 4 | 85 (51.5) | 24 (35.8) | 109 (47.0) |
| 5 | 28 (17.0) | 17 (25.4) | 45 (19.4) |
| ≥6 | 17 (10.3) | 9 (13.4) | 26 (11.2) |
| Parental marriage status | | | |
| Divorced | 18 (10.9) | 8 (11.9) | 26 (11.2) |
| Separated | 6 (3.6) | 1 (1.5) | 7 (3.0) |
| Married | 131 (79.4) | 54 (80.6) | 185 (79.7) |
| Family happiness | | | |
| Very unpleasant | 7 (4.2) | 9 (13.6) | 16 (6.9) |
| Unpleasant | 18 (10.9) | 9 (13.6) | 27 (11.6) |
| General | 63 (38.2) | 22 (33.3) | 85 (36.8) |
| Pleasant | 53 (32.1) | 23 (34.8) | 76 (32.9) |
| Very pleasant | 24 (14.5) | 3 (4.5) | 27 (11.7) |
| SSF | | | |
| Yes | 19 (11.5) | 8 (11.9) | 27 (11.6) |
| No | 145 (87.9) | 59 (88.1) | 204 (87.9) |

SSF = Social Security Fund.

With specific reference to those participants who obtained higher scores on the CPYDS in the post-test than the pretest, there were 94 (56%) and 29 (47%) participants in School A and School B, respectively. However, in School A, there was still a slightly significant increase in behavioral intention to participate in gambling from pre- to post-tests and to drink alcohol in School B. Overall, among all the 232 participants of School A and School B, 123 (53%) of them had improvement. The results showed that there was significant improvement on the total scale of the CPYDS and the two composite scores (Table 3). However, a slight tendency in behavioral intention for alcohol drinking is still present.

**TABLE 2**
**Changes in the Program Participants Based on the CPYDS, LIFE, and BI Scales**

| | Pretest | | Post-Test | | *t* Value | *p* Value |
|---|---|---|---|---|---|---|
| | Mean | SD | Mean | SD | | |
| School A (n = 165) | | | | | | |
| CPYDS | 4.34 | 0.54 | 4.36 | 0.51 | -0.511 | 0.610 |
| SC subscale | 4.21 | 0.76 | 4.35 | 0.68 | -2.790 | **0.006** |
| EC subscale | 4.00 | 0.84 | 4.19 | 0.87 | -3.014 | **0.003** |
| CC subscale | 4.25 | 0.76 | 4.42 | 0.75 | -2.714 | **0.007** |
| SD subscale | 4.60 | 0.77 | 4.74 | 0.73 | -2.256 | **0.025** |
| PD Score | 4.23 | 0.54 | 4.31 | 0.52 | -2.289 | **0.023** |
| PC Score | 4.24 | 0.60 | 4.37 | 0.54 | -3.169 | **0.002** |
| LIFE | 3.96 | 0.94 | 3.76 | 1.02 | 2.659 | **0.009** |
| BI | 1.34 | 0.46 | 1.40 | 0.45 | -2.200 | **0.029** |
| BI(item 5) | 1.24 | 0.56 | 1.43 | 0.79 | -3.014 | **0.003** |
| School B (n = 67) | | | | | | |
| CPYDS | 4.33 | 0.49 | 4.22 | 0.76 | 1.680 | 0.098 |
| SE subscale | 3.29 | 0.58 | 3.63 | 0.94 | -2.696 | **0.009** |
| LIFE | 3.92 | 1.11 | 3.46 | 1.11 | 2.966 | **0.004** |
| BI | 1.52 | 0.66 | 1.65 | 0.65 | -1.786 | 0.079 |
| BI (item 1) | 1.95 | 1.09 | 2.27 | 1.16 | -2.828 | **0.006** |
| Total (n = 232) | | | | | | |
| CPYDS | 4.34 | 0.52 | 4.32 | 0.59 | 0.555 | 0.579 |
| SC subscale | 4.25 | 0.75 | 4.37 | 0.73 | -2.597 | **0.010** |
| LIFE | 3.95 | 0.99 | 3.68 | 1.05 | 3.923 | **0.000** |
| BI | 1.37 | 0.53 | 1.47 | 0.53 | -2.836 | **0.005** |
| BI (item 1) | 1.87 | 0.98 | 2.07 | 1.06 | -2.897 | **0.004** |
| BI (item 5) | 1.30 | 0.68 | 1.47 | 0.81 | -3.088 | **0.002** |

*Note*: CPYDS = mean of the 15 subscales. SC = Social Competence subscale; EC = Emotional Competence subscale; CC = Cognitive Competence subscale; SD = Self-Determination subscale; SE = Self-Efficacy subscale; PD (Personal Development Score) = mean of 10 subscales excluding Bonding, Prosocial Involvement, Prosocial Norms, Spirituality, and Recognition for Positive Behavior subscales. PC (Psychosocial Competence Score) = mean of social, emotional, cognitive, behavioral, and moral competencies subscales. BI (item 1) = Will you drink alcohol in the coming 2 years? BI (item 5) = Will you participate in gambling activities in the coming 2 years? Significant *p* values are in bold.

## Factors Related to Youth Growth

Based on the general linear model, data on gender, age, financial status, parents' marital status, and happiness of family life were collected and used as confounding factors in the analysis of difference between pre- and post-test outcome variables (CPYDS). One of the factors, happiness of family life, was found to have a significant difference in the score of the CPYDS ($p = 0.001$). When correlation was used to analyze the relationship with the CYPDS post-test score, a positive relationship ($\gamma_s = 0.193$, $p = 0.000$) with the score, i.e., the happier the family life, the higher the score of the CPYDS, was made manifest. Apart from these, the LIFE scale also displayed a positive relationship with the CPYDS ($\gamma = 0.479$, $p = 0.000$).

**TABLE 3**
**Participants having Positive Changes on the CPYDS Scale**

| | Pretest | | Post-Test | | *t* Value | *p* Value |
|---|---|---|---|---|---|---|
| | Mean | SD | Mean | SD | | |
| School A (n = 94) | | | | | | |
| CPYDS | 4.11 | 0.44 | 4.45 | 0.49 | -11.921 | **0.000** |
| PD | 4.01 | 0.44 | 4.41 | 0.49 | -12.548 | **0.000** |
| PC | 4.01 | 0.50 | 4.46 | 0.52 | -10.538 | **0.000** |
| LIFE | 3.73 | 0.92 | 3.86 | 0.95 | -1.339 | 0.184 |
| BI | 1.35 | 0.47 | 1.45 | 0.47 | -1.994 | **0.049** |
| BI (item 5) | 1.25 | 0.60 | 1.42 | 0.78 | -2.316 | **0.023** |
| School B (n = 29) | | | | | | |
| CPYDS | 4.38 | 0.43 | 4.70 | 0.48 | -7.331 | **0.000** |
| PD | 4.29 | 0.43 | 4.65 | 0.50 | -7.190 | **0.000** |
| PC | 4.42 | 0.57 | 4.68 | 0.59 | -3.230 | **0.003** |
| LIFE | 4.13 | 0.96 | 4.16 | 0.80 | -0.161 | 0.873 |
| BI (item 1) | 1.80 | 1.01 | 2.17 | 1.15 | -2.009 | **0.054** |
| Total (n = 123) | | | | | | |
| CPYDS | 4.18 | 0.45 | 4.52 | 0.49 | -13.990 | **0.000** |
| PD | 4.08 | 0.45 | 4.47 | 0.50 | -14.483 | **0.000** |
| PC | 4.11 | 0.54 | 4.51 | 0.54 | -10.572 | **0.000** |
| LIFE | 3.83 | 0.94 | 3.93 | 0.92 | -1.186 | 0.238 |
| BI | 1.37 | 0.51 | 1.48 | 0.51 | -2.209 | **0.029** |
| BI (item 1) | 1.93 | 1.01 | 2.12 | 1.02 | -2.040 | **0.044** |

*Note*: CPYDS = mean of the 15 subscales. PD (Personal Development Score) = mean of 10 subscales excluding Bonding, Prosocial Involvement, Prosocial Norms, Spirituality, and Recognition for Positive Behavior subscales. PC (Psychosocial Competence Score) = mean of social, emotional, cognitive, behavioral, and moral competencies subscales. BI (item 1) = Will you drink alcohol in the coming 2 years? BI (item 5) = Will you participate in gambling activities in the coming 2 years? Significant *p* values are in bold.

The general linear model was also performed separately in two schools; gender was found to be a confounding factor in School A. Male students had positive changes; the average CYPDS score of male students increased, while female scores did not ($p = 0.013$). In School B, the happiness of family life was the confounding factor ($p = 0.000$).

## Findings of the Focus Group Interviews

Ten students from School A participated in one group interview, four were males and six were females. Three of them were very quiet and seldom responded to the interviewer. Six students from School B participated in another group interview; all of them were male and were responsive to the group discussion. The qualitative findings were mainly analyzed in two areas: the general impression of the program and the perceived benefits of the program to themselves. In order to examine the reliability of the coding, both the first author and the research assistant were involved in the data analysis, but separately. The inter-rater reliability was 0.90. Regarding the general impression, the preliminary analyses were classified into positive and negative comments of the program. Among the 10 who gave feedback, five claimed “feeling bored” and the rest described the program as better than their moral class by “learning more”, “not so boring”, “interesting”, and they “learned how to interact with others”. The activities that aroused their interests were discussion and video. Some narratives of students were as follows:

> “A fresh feeling, different from the formal moral class, I can understand more and learn how to mix with others.” - From a male student of School B.

> “By watching the video, I would understand more about the experiences of others, be ready for future life, for example, I have been alerted to the danger of drug addiction, learned to draw upon the merits of others and at the same time avoid repeating the faults of others.” - From a male student of School A.

With reference to the perceived benefits, most benefits could be classified into the personal level, such as improvement of one’s emotion, and interpersonal level, such as enhanced interpersonal skills. Nine students with positive feedback asserted that after the program, they started to have “more control in one’s emotion”, to “become more understanding to others”, to “know how to interact with others”, to “learn from other’s faults”, and to “have more self-confidence” (four of them). There was also a small increase in the sense of responsibility. Some narratives of students were as follows:

> “Concerning my emotion, it is not so easy now for me to lose my temper. I try to think from the perspective of others. I am not so impulsive now and it has helped me control my emotion and I practice what I learn in my daily life.” - From a female student of School A.

> “There is a change of my personality, I know how to improve and accept others.” - From a female student of School A.

> “I am no longer that lazy. I never submitted all my homework before. After completing the lesson on responsibility, at present, I have only failed to submit my homework 2 times a week.” - From a male student of School B.

> “My self-confidence was enhanced by my teacher during these few lessons. He encouraged us not to give up, do not leave things half done.” - From a male student of School B.

Some suggestions such as “control of class discipline” and “more lively presentation” were also received.

## DISCUSSION

Regarding the objective outcome evaluation, significant differences between pre- and post-tests were found in several subscales and the composite scores in School A. They are related to the Personal Development and Psychosocial Competence that are important components for youth growth. There may be several possible reasons why these competences are present in School A. First, the program implementers are experienced social workers who may be more familiar with interactive activities. Second, School A is of a strong Christian background, which may have some positive effects on the students in moral education. Finally, the general family background of the participants may also affect their personal changes. More students in School A, 78 (46.6%), expressed family happiness than in School B, 26 (39.3%). On the other hand, only one significant positive change was found in School B. Some possible reasons follow. First, the program was run by new teachers who may not have been accustomed to informal teaching. A study by Shek and Sun[22] found that one of the difficulties encountered in program implementation by teachers is that it is difficult for them to handle poor classroom discipline. Second, the school was without any religious background and the general academic performance of participants may be lower as compared with that of School A. However, the significant change of Self-Efficacy highlighted the effectiveness of the program in this special area of improved skills for coping and mastery, which were consistent with some feedback from the participants of School B in the focus group interview. When seeing the two schools together, significant positive change is found in the Social Competence subscale, which is also an important indicator that has to be developed in Secondary 1 Students, especially for those who fail to be promoted in their original primary school and who then have to join a new school.

As far as a slight increase in the tendency of the problem behaviors concerned, one of the possible reasons why School A students had an increase in gambling intentions may be due to the influences of family members. With the booming of the gaming business in Macau, increasing numbers of family members who work directly or indirectly with gambling activities may make students more receptive to gambling, while School B students had an increase in drinking intentions that may be due to the differences of their demographic background. Most students in School B are male and older in age. Alcohol drinking can be a symbol of showing masculinity and maturity, especially for those whose academic performance is poor. With regard to the decrement in life satisfaction, there are several possible reasons. Involuntary change to one’s unflavorable secondary school due to poor academic results may be one reason. Stress in making new friends when changed to secondary school, and adapting to the new way of learning with more homework and higher academic performance expectations may be other reasons. This issue warrants the attention of the school administration.

With specific reference to the percentage of participants who got higher scores on the CPYDS, 53% of the participants had improvement with significant increases in the total scale of the CPYDS and the two composite scores. Suggested by Shek and Ma[15], at least 20% of students may have greater psychosocial needs at each grade and need a Tier 2 program; therefore, as a pilot study, the program effect in Macau could be regarded as encouraging, although the intervention is of a short duration. However, the slight increase in the behavior intention of drinking alcohol and participation in gambling activities deserves our attention. A study by Lou and Shek[23] found that adolescent drinking is an emergent problem in Hong Kong. Since Hong Kong and Macau are neighboring cities and share a similar youth culture, more action should be taken to prevent adolescent drinking, which is becoming a serious problem. Relating to gambling, an opinion study by Lui and Lau[24] on youth perception of the influences upon Macau after the opening of the gaming licensure showed that adolescents were more willing to work in the gaming industry and its related businesses. With the booming of the economy led by the gaming business, adolescents are more receptive to participation in gambling activities. Another study by the Anglican Macau Social Services[25] found that the older the youth, the greater the participation in soccer gambling in Macau. Therefore, greater effort should be put into preventing adolescents who lack precaution from becoming addicted to gambling.

As far as the factors related to youth growth are concerned, since happiness of the family was found to be the most significant factor affecting the growth of the adolescents of both schools, more collaboration should be made from school teachers and school social workers to help those students who have family problems that affect their holistic growth. Since the LIFE scale was also found to have a positive relationship with the CPYDS, it is again shown to be a good indicator for measuring youth growth and can be used as an outcome measure simultaneously with other youth development measurements. However, as participants showed a decrease in the LIFE scale, it warrants further study to explain why Secondary 1 students are not satisfied with their quality of life. Is it related to adaptation to a new environment including venue, teachers, curriculum, or changes due to adolescent growth? Relating to gender, which was found to be a confounding factor in School A, one of the reasons why male students have more changes than female students may be that they are more ready to build up personal and psychosocial competence to show masculinity and attractiveness to female students.

Regarding the general impression of the program from the focus group interviews, some of the students perceived the program to be boring; however, when compared with their conventional moral or civic classes, the program was more interesting because it had discussions and video shows. More peer interaction among the students in the class as shown in the significant increase of the score in the Social Competence subscale of the CPYDS demonstrated one aspect of the effectiveness of the program even though some of the students perceived it in a negative way. Observations from Shek[26] showed that approximately 15% of the participants failed to perceive the program as effective. It is interesting to note that even though the class may give a boring impression to some students, more students still learned and benefitted from the program. With reference to the perceived benefits of the program, all participants were positive in their feedback. Generally speaking, benefits in both personal level and interpersonal level were observed, which is consistent with the findings by Shek and Ng[13]. The focus group observations are generally consistent with the objective outcome evaluation findings in this study, i.e., that the students changed in a positive direction in various developmental domains. However, constructive feedback on the control of classroom discipline and more lively presentation by program implementers should be addressed when improving the implementation of the program for the next group of students.

Based on the findings from the objective outcome evaluations and the focus group interviews, it can be concluded that there is positive evidence supporting the effectiveness of the adopted and modified Tier 1 Program of the Hong Kong Project P.A.T.H.S. In addition, special attention should be paid to the behavioral intention of alcohol drinking and participation in gambling activities.

Although the present findings provide some evidences illustrating the program effectiveness of the modified Project P.A.T.H.S. in Macau, there are several limitations of the study. First, the positive impact of the program is comparatively small as compared with the effects in Hong Kong. One of the reasons may be the lack of readiness of the program implementers and the students in Macau. Only two schools were willing to join the pilot study. More promotion should be made to other schools and additional training should be provided for the potential program implementers. However, the implementation of the Secondary 2 program was better received after the successful trial of the Secondary 1 program. Second, the time for the implementation of the program is still short, only 10 h for both schools. A follow-up study is continued for the pilot schools in the Secondary 2 program to measure the long-term effects after collecting the longitudinal data next year. Third, as it is a pre- and post-test pre-experimental study without a control group, a more rigorous result cannot be obtained. Despite these limitations, the present study provides the groundwork for further exploration of the effectiveness of the modified Tier 1 Program of the Project P.A.T.H.S. on the holistic development of Chinese adolescents in Macau.

## ACKNOWLEDGMENTS

The research team would like to thank Professor Daniel Shek and his research team in Hong Kong for their support in launching this study in Macau. Furthermore, this study was financially supported by the Education and Youth Bureau of the Macau Government.
